# Oncometabolite signatures from tumor-stroma crosstalk as potential non-invasive biomarkers

**DOI:** 10.1038/s41420-026-03172-1

**Published:** 2026-05-22

**Authors:** Alessia Parascandolo, Maria C. Magnifico, Emanuele De Vita, Peiman Hematti, Cosimo Distante, Francesco Corcione, Marco Varelli, Francesca Cutruzzolà, Alberto Macone, Mikko O. Laukkanen

**Affiliations:** 1 Department of Translational Medical Sciences, University of Naples Federico II, Naples, Italy; 2 Department of Biochemical Sciences “Alessandro Rossi Fanelli”, Sapienza University of Rome, Laboratory affiliated to the Istituto Pasteur Italia-Fondazione Cenci Bolognetti, Rome, Italy; 3 Istituto Varelli, Naples, Italy; 4 Department of Medicine, Division of Hematology/Oncology, Medical College of Wisconsin, Milwaukee, WI USA; 5 Institute of Applied Sciences and Intelligent Systems (ISASI), National Research Council (CNR), Lecce, Italy; 6 Clinica Mediterranea, Naples, Italy; 7 Department of Biochemical Sciences “Alessandro Rossi Fanelli”, Sapienza University of Rome, Rome, Italy

**Keywords:** Mechanisms of disease, Cancer metabolism, Diagnostic markers

## Abstract

Tumor metabolism, a crucial component in cancer progression, represents a potential prognostic and diagnostic platform in cancer detection. Here, we show that patient-specific stromal mesenchymal cells exhibit distinct behaviors in promoting either tumor growth or dissemination. Tumor-associated fibroblasts (TAFs) isolated from non-metastasized colorectal adenocarcinomas predominantly supported cancer cell proliferation, whereas TAFs isolated from metastatic adenocarcinomas facilitated cancer cell migration. An in vitro analysis of stromal paracrine factors revealed that variations in mitochondrial activity and the secretion of specific metabolites were closely associated with distinct tumor-stroma interactions. Among the oncometabolites identified, we validated amino acid expression in urine samples from 19 colon cancer patients to assess their potential as diagnostic biomarkers. Our results showed patient-specific alterations in oncometabolite expression, which were significantly different from those of healthy control individuals. The specificity, sensitivity, and accuracy analysis indicated 93–100% specificity, 74–82% sensitivity, and 84–89% accuracy of single metabolites in distinguishing cancer patients from healthy controls. While no false negatives were observed, urine samples from nine patients with various inflammatory conditions (including diverticulitis, appendicitis, and chronic gastritis) yielded false positives. Sensitivity analysis and the t-Distributed Stochastic Neighbor Embedding (t-SNE) nonlinear dimensionality reduction technique revealed distinct metabolite profiles for healthy controls, colon cancer patients, and patients diagnosed with inflammation. Overall, our findings suggest that the identified oncometabolites, when integrated into a biomarker panel, hold promise as a novel non-invasive tool for screening individuals at risk of cancer and inflammatory malignancies.

## Introduction

Colorectal cancer (CRC) is one of the leading causes of cancer-related mortality worldwide. Despite current stool-based and colonoscopy screening procedures, or imaging techniques, such as computed tomography (CT), magnetic resonance imaging (MRI), and positron emission tomography (PET), detection of CRC remains a challenge due to the lack of minimally distressing diagnostic methods. While fecal DNA and blood cell analyses have demonstrated sensitivity rates up to 95% and specificity rates of 82%, they are limited by their inability to reliably differentiate between benign adenomas, localized adenocarcinomas, and metastatic disease [1, 2]. More importantly, the sensitivity and specificity are affected by other pathologies including inflammatory bowel disease, ischemic colitis, bacterial infections, diverticular disease, peptic ulcer, and tissue damage, which results in fecal blood cell contamination.

In recent years, attention has shifted toward the role of the tumor microenvironment (TME) in shaping cancer progression and therapeutic outcomes [3–9]. Growth factors, cytokines, metabolites, and other paracrine factors secreted by the stroma represent a crucially important cluster of messengers, determine the characteristics of the TME, and have potential as diagnostic biomarkers [10, 11]. In addition, metabolic reprogramming and metabolites, which have a unique composition in each phase of tumorigenesis, have been identified as part of a metabolic signature in CRC. Specific metabolic intermediates, detectable from patient’s urine, saliva, and breath using advanced detection technologies, have potential as diagnostic biomarkers [10–13]. In this study, we investigated the impact of paracrine secretion from colon adenocarcinoma TAFs on cancer cell function and identified metabolic intermediates, which could hold potential for use in the diagnosis of colon adenocarcinoma.

## Results

### The patient-specific effect of tumor stroma TAFs on cancer cell DNA replication

**Fig. 1:**
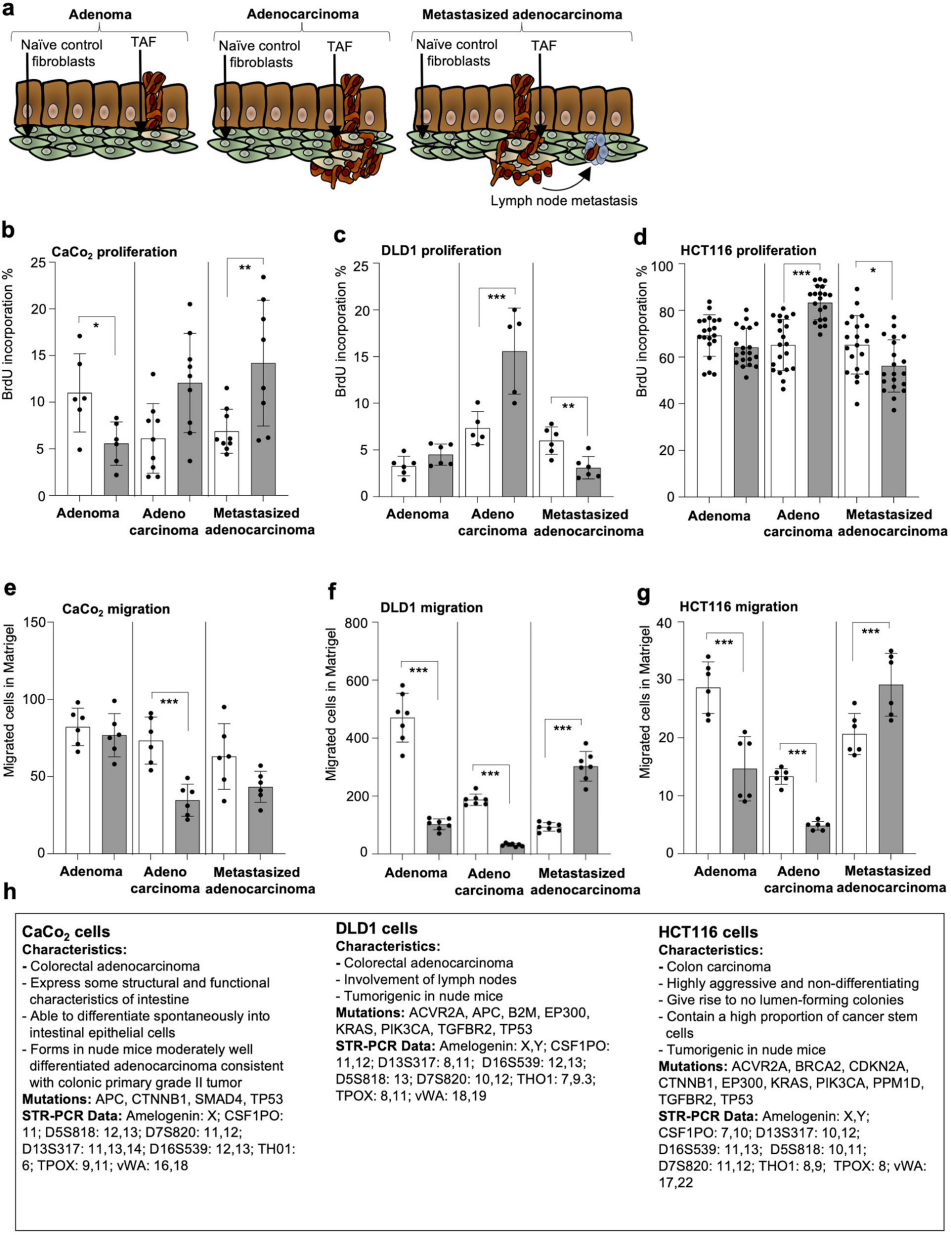
DNA replication and migration of cancer cells in stromal cell co-culture. White bar graphs refer to naïve fibroblasts, grey bar graphs refer to TAFs. TAFs and their naïve fibroblast counterparts were isolated from colon adenoma, adenocarcinoma, and metastasized adenocarcinoma patients. **a** Sketch of in vitro model. **b**–**d** BrdU incorporation into CaCo_2_, DLD1, and HCT116 colon cancer cell DNA in co-culture with colon fibroblasts. **e**–**g**. Chemotactic migration of CaCo_2_, DLD1, and HCT116 cells through an extracellular matrix towards naïve colon fibroblasts and TAFs. **h** Characteristics of CaCo_2_, DLD1, and HCT116 colon cancer cells. Statistical analysis: The *p*-values were indicated as follows: *p < 0.05, **p < 0.01, ***p < 0.001.

To assess the growth-promoting effects of colon tumor-derived TAFs, we co-cultured CaCo_2_, DLD1, and HCT116 colon cancer cells with fibroblasts isolated from patients with benign colon adenoma, non-metastasized adenocarcinoma, and metastasized adenocarcinoma (Fig. 1a–d). TAFs derived from benign adenomas exhibited downregulation (50.5%, *p* < 0.05) in BrdU incorporation in CaCo_2_ cells, whereas they had a comparable effect on BrdU incorporation into DLD1 and HCT116 cancer cell DNA as their naïve fibroblast counterparts. TAFs isolated from non-metastasized adenocarcinoma patient increased BrdU incorporation in all cell cancer lines, while TAFs from metastasized adenocarcinoma displayed a reduction both in DLD1 (52%, *p* < 0.01) and in HCT116 cells (13.8%, *p* < 0.05) in their capacity to support DNA replication, suggesting a diminished ability of TAFs from metastatic tumors to foster tumor mass expansion. Notably, TAFs derived from metastasized disease increased BrdU incorporation (48.6%, *p* < 0.01) in CaCo_2_ cells (Fig. 1b–d).

### The patient-specific effect of tumor stroma on cancer cell migration

Intratumoral cancer cell migration is one of the earliest steps in metastasis, a process increasingly recognized as being coordinated by tumor stroma. Therefore, next we studied the patient-specific effect of colon TAFs on cancer cell migration in vitro. The migration of CaCo_2_ cells towards TAFs isolated from colon adenoma showed no significant difference compared with naïve counterparts, whereas migration of DLD1 and HCT116 cells toward adenoma-derived TAFs was reduced by 78.1% (*p* < 0.001) and 53.1% (*p* < 0.001), respectively, compared with migration toward naïve counterpart fibroblasts (Fig. 1e–g).

Fibroblasts isolated from a non-metastasized colon adenocarcinoma patient induced 72.2% (*p* < 0.001), 83.2% (*p* < 0.001), and 63.7% (*p* < 0.001) decrease in migration of CaCo_2_, DLD1, and in HCT116 cells, respectively, toward TAFs compared with naïve fibroblasts. In contrast, TAFs from metastasized adenocarcinoma exhibited a non-significant 31.5% decrease in CaCo_2_ cell migration, while exerting a pronounced pro-migratory effect on DLD1 and HCT116 cells, increasing migration by 69.1% (*p* < 0.001) and 37.7% (*p* < 0.001), respectively, compared with naïve counterpart fibroblasts (Fig. 1e–g). Current findings underscore the heterogeneity of stromal influence on cancer cell behavior, demonstrating that stromal cells from different patients may preserve certain clinical characteristics within in vitro cultures.

### Growth factor, cytokine expression pattern, and related signal transduction pathway analysis

**Fig. 2:**
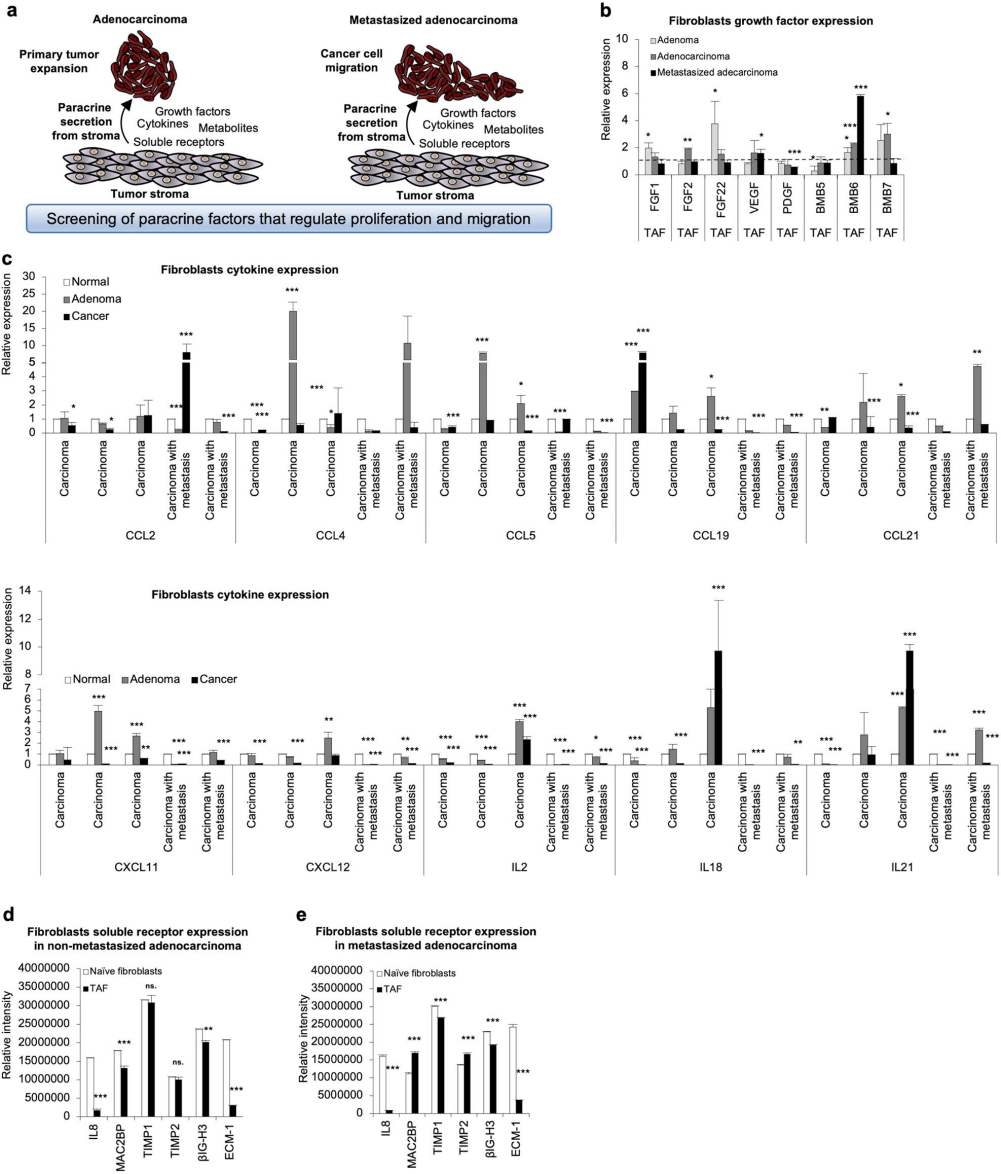
Expression profiling. **a** Growth factors, cytokines, and soluble receptors were analyzed from colon naïve fibroblasts and their counterpart TAFs. **b** Growth factor expression in colon fibroblasts. Expression levels of growth factors (*FGF1*, *FGF2*, *FGF22*, *VEGF*, *PDGF*, *BMP5*, *PMP6*, and *BMP7*) were assessed by qPCR in naïve fibroblasts and TAFs isolated from colon adenomas, non-metastasized adenocarcinoma, and metastasized adenocarcinomas. The hatched line shows the naïve fibroblast value. **c** Cytokine expression in colon naïve fibroblasts, and in fibroblasts from adenoma and adenocarcinoma-derived TAFs was performed using qPCR. The analysis showed stage- and patient-specific differences in cytokine expression, including variations in common cytokines that influence cell growth and migration. **d** Soluble receptor profiling in non-metastatic adenocarcinoma. **e** Soluble receptor profiling in metastatic adenocarcinoma. Statistical analysis: Data are expressed as mean standard deviation (SD). Statistical significance is indicated as *p < 0.05, **p < 0.01, ***p < 0.001.

To investigate the common factors involved in the transition of primary tumors to metastatic disease, we examined the growth factor and cytokine mRNA expression profiles (Fig. 2a–c). Among growth factors analyzed, only *BMB6* expression demonstrated a gradual increase in TAFs compared with naïve fibroblast counterparts that. However, this change alone is unlikely to explain disease progression [14]. The cytokine analysis suggested downregulation of *CCL19*, *CXCL11*, *CXCL12*, *IL2*, *IL18*, and *IL21* in TAFs isolated from the metastatic patient. The function of these cytokines in carcinogenesis remain incompletely understood and may depend on interactions with the function other factors and their corresponding receptors [15–20].

A screening of additional paracrine factors secreted from fibroblasts revealed a downregulation of IL8, βIG-H3, and ECM-1 in both non-metastasized and metastasized cancer contexts. Additional markers, such as MAC2βBP, TIMP1, and TIMP2 displayed variable expression profiles (Fig. 2d, e, Fig. Supplemental Fig. S1a, b). Although growth factors, cytokines, and soluble receptors play essential roles in the carcinogenic process, the expression patterns of the molecules investigated in this study do not provide definitive insight into the regulatory functions of stromal cells in influencing tumor mass expansion and metastatic dissemination.

We performed signal transduction analysis of stromal fibroblasts to dissect the roles of small GTPase RAS, RAC, and MAPK pathways, which are implicated in colon cancer progression [21, 22]. The pathway activation studies corroborated the expression analysis indicating minor or no differences in the activation of MAP kinases in fibroblasts isolated from normal colon, non-metastasized adenocarcinoma, or metastasized adenocarcinoma (Supplemental Fig. S2a). To study the cell membrane associated cytoplasmic signaling in fibroblasts, we performed RAS and RAC pulldown analysis in HCT116 cells, which demonstrated similar small GPTase activation levels in cancer cells grown at the presence of naïve fibroblasts and TAF (Fig. S2b, c). The signaling molecule activation analysis of JNK, SMAD, GSK3β, β catenin, YAP TAZ, PKA substrates, and CREB from patient’s colon normal tissue, adenoma tissue, and adenocarcinoma tissue both from non-metastasized adenocarcinoma and metastasized adenocarcinoma further corroborated the conclusion of similar signaling cascade activation in cancer tissues (Fig. S2d).

### Analysis of mitochondrial function

**Fig. 3:**
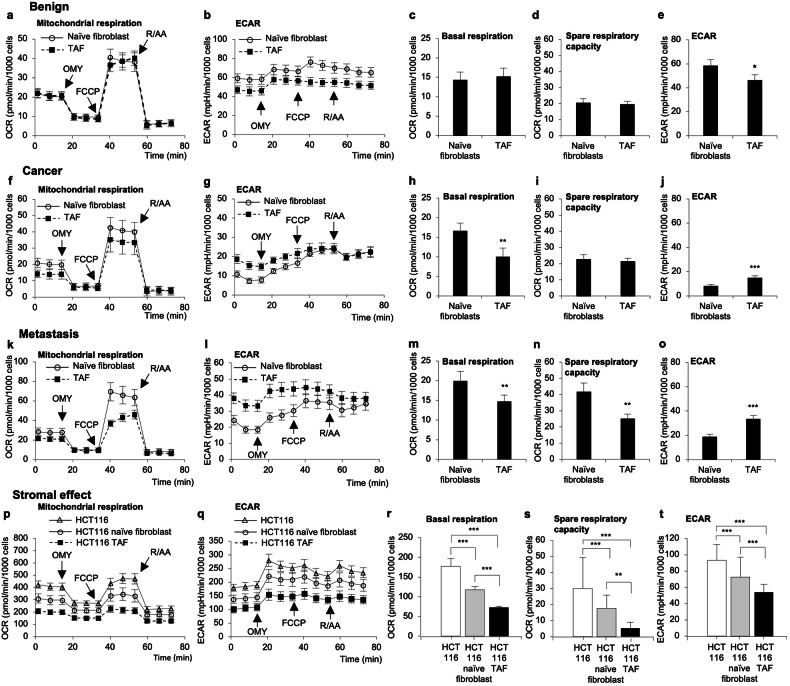
Mitochondrial respiration analysis of colon naïve fibroblasts and TAFs from benign and malignant colon tumors. **a**–**e** Fibroblasts isolated from a patient with a benign tumor showed modest alterations in ECAR. **f**–**j** Fibroblasts from a non-metastasized colon adenocarcinoma exhibited significantly reduced basal respiration and markedly increased ECAR. **k**–**o** Fibroblasts derived from a metastatic colon adenocarcinoma demonstrated significantly lower basal respiration, reduced spare respiratory capacity, and elevated ECAR. **p**–**t** Mitochondrial respiration in HCT116 cells cultured in the condensed medium from naïve fibroblasts or TAFs isolated from benign tumors revealed a significant decrease in basal respiration, spare respiratory capacity, and ECAR in response to naïve fibroblast-derived medium. These parameters were further diminished in the presence of TAF-derived medium. Statistical analysis: The analysis was done in triplicates. Data are presented as mean standard deviation (SD). Significance is indicated by *p < 0.05, **p < 0.01, ***p < 0.001.

Metabolic reprogramming of the tumor microenvironment and increased mitochondrial activity have been shown to promote fibroblast maturation into cancer-associated fibroblasts, as well as cancer cell proliferation and metastasis [6, 23]. Therefore, we examined the bioenergetic profiles of fibroblasts isolated from patients with benign adenoma, non-metastasized adenocarcinoma, and metastasized adenocarcinoma to investigate the contribution of metabolism to the transition from primary tumors to metastatic disease. Our analysis of TAFs derived from benign adenomas, in comparison to their naïve fibroblast counterparts, revealed comparable levels of mitochondrial basal respiration, which is indicative of the oxygen consumption necessary for ATP production, and spare respiratory capacity, reflecting the cells’ capacity to respond to increased energy demands. The extracellular acidification rate (ECAR) was significantly reduced (*p* < 0.05) in TAFs relative to naïve fibroblasts (Fig. 3a–e). In TAFs isolated from non-metastasized adenocarcinoma, we observed a significant (*p* < 0.01) decrease in basal respiration compared to controls, while spare respiratory capacity remained similar. Furthermore, ECAR, was significantly elevated (*p* < 0.001) in these TAFs (Fig. 3f–j). In TAFs from metastasized adenocarcinoma, we noted significant reduction in both basal respiration (*p* < 0.01) and spare respiratory capacity (*p* < 0.01), alongside a marked increase in ECAR (*p* < 0.001) (Fig. 3k–o).

Lastly, we studied the paracrine effects of fibroblasts derived from benign tumors on cancer cells. The conditioned medium from naïve fibroblasts significantly (*p* < 0.001) reduced basal respiration in HCT116 cells compared to those cultured in their own medium. Furthermore, HCT116 cells exposed to the conditioned medium from adenoma TAFs exhibited an even more pronounced decrease in basal respiration, demonstrating significantly (*p* < 0.001) lower levels compared to both HCT116 cells in their own medium and those cultured with naïve fibroblast-conditioned medium. Similarly, HCT116 cells displayed significantly lower basal respiration, decreased spare respiratory capacity, and reduced ECAR when exposed to the conditioned medium from both naïve fibroblasts and adenoma TAFs relative to their own culture medium (Fig. 3p–t).

### Analysis of oncometabolites in fibroblasts cultures and in patient urine samples

**Fig. 4:**
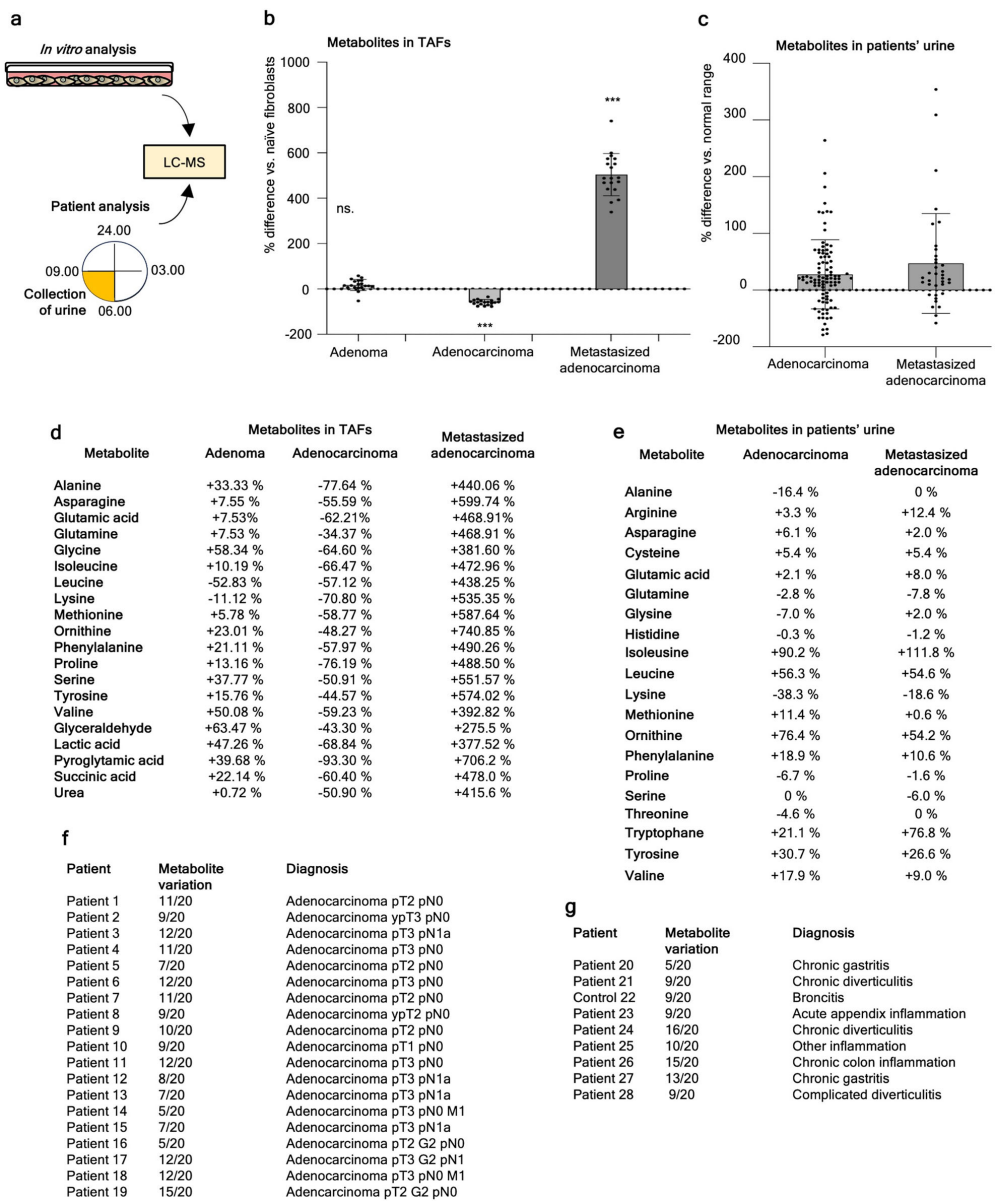
Oncometabolite analysis of colon fibroblasts and patient urine samples. **a** Metabolites secreted to fibroblast culture medium and patient urine were analyzed with liquid chromatography mass spectrometry. All urine samples were collected between the hours 06.00–09.00 to avoid alterations caused by the circadian rhythm. **b** The average concentrations of the secreted metabolites in vitro. The data demonstrates a non-significant 35.4% (SD = 30) increase in secretion from TAFs derived from adenoma, a significant (*p* < 0.001) 58.5% (SD = 13.9) decrease in the amount of oncometabolites in the medium of TAFs isolated from non-metastasized fibroblasts, and a significant (*p* < 0.001) 495.2% (SD = 112.2) increase in the concentration of oncometabolites in medium of TAFs isolated from metastasized fibroblasts as compared to their naïve counterpart fibroblasts. The hatched line shows the naïve fibroblast value. **c** The average concentrations of the secreted metabolites in urine samples. The hatched line shows the healthy control values. The analysis of all metabolites demonstrated a non-significant overall difference between controls and patients. **d** Itemized variation in metabolite expression in TAFs compared to naïve counterpart fibroblasts. **e** Itemized variation analysis in the amino acid expression in urine samples as compared to normal controls shows marked increases in the expression of isoleucine, leucine, ornithine, tryptophan, and tyrosine in both non-metastasized and metastasized cancer. **f** The metabolite variation of amino acid expression in urine samples of 19 colon adenocarcinoma patients, including 7 patients with metastasis. The metabolite variation column shows the number of abnormally expressed metabolites out of the total number of 20 amino acids measured. **g** The patient cohort contained 9 individuals with different inflammations, which affected the metabolite expression. Statistical analysis: Data are presented as mean standard deviation (SD). Significance is indicated by *p < 0.05, **p < 0.01, ***p < 0.001.

To identify metabolites associated with alterations in mitochondrial activity, we performed liquid chromatography-mass spectrometry analysis of culture media and patient urine samples (Fig. 4a). In comparison to naïve fibroblasts, the medium collected from TAFs derived from adenomas exhibited a non-significant increase in average metabolite concentrations. Intriguingly, TAFs from non-metastasized adenocarcinoma demonstrated a significant decrease in metabolites secretion compared to their naïve counterparts, while TAFs isolated from metastasized adenocarcinoma displayed a remarkable increase in metabolite secretion (Fig. 4b).

To validate the in vitro data in patients, we analyzed a metabolite fingerprint, a panel of 20 amino acids, in urine samples. We recruited 15 healthy control persons, 19 colon cancer patients, and 9 patients diagnosed with inflammation to assess the potential impact of inflammation on false-positive diagnoses [24]. In contrast to findings in fibroblast culture systems, where oncometabolite secretion was either increased or decreased in non-metastatic versus metastatic cancer cells, our urinary analysis revealed a more stochastic pattern of amino acid expression (Fig. 4c).

The analysis of individual metabolites in fibroblasts revealed eight aberrantly expressed non-essential amino acids (alanine, asparagine, glutamic acid, glutamine, glycine, proline, serine, and tyrosine), six essential amino acids (isoleucine, leucine, lysine, methionine, phenylalanine, and valine), and one non-protein amino acid (ornithine) (Fig. 4d). The reduced production of lactic acids, −68.84% in non-metastasized adenocarcinoma and 377.52% increase in metastasized adenocarcinoma, aligns with mitochondrial respiration analyses, which indicated decreased ATP production via oxidative phosphorylation and increased ECAR in TAFs derived from metastatic adenocarcinoma. Other metabolites markedly altered in tumor stroma included glyceraldehyde (−43.3%), pyroglutamic acid (93.3%), succinic acid (−60.4%), and urea (−50.9%) in fibroblasts isolated from non-metastasized cancer. In metastasized adenocarcinoma the corresponding values were glyceraldehyde (275.5%), pyroglutamic acid (706.2%), succinic acid (478.0%), and urea (415.6%) (Fig. 4d).

Analysis of individual metabolites in urine samples revealed a largely stochastic expression pattern of amino acid expression. Nonetheless, significant deviations from the control group were observed, most notably in the levels of isoleucine, leucine, ornithine, tryptophan, and tyrosine, which were markedly elevated in both non-metastatic and metastatic cancer cases (Fig. 4e). The overall metabolite variation pattern in 19 colon adenocarcinoma patients showed expression changes in 5 to 15 amino acids across patients diagnosed with stage pT1 to pT3 colon adenocarcinoma, grade G2, with or without lymph node or hepatic metastasis (Fig. 4f).

To investigate false positives associated with inflammation, we recruited nine patients diagnosed with various inflammatory conditions. The false positives showed variation range from 5 to 16 abnormally expressed amino acids out of 20 measured amino acids (Fig. 4g), further corroborating previously reported overlapping metabolic aberrations in tumorigenesis and immune defense [24–26].

### Specificity, sensitivity, and accuracy of metabolite expression in urine samples

**Fig. 5:**
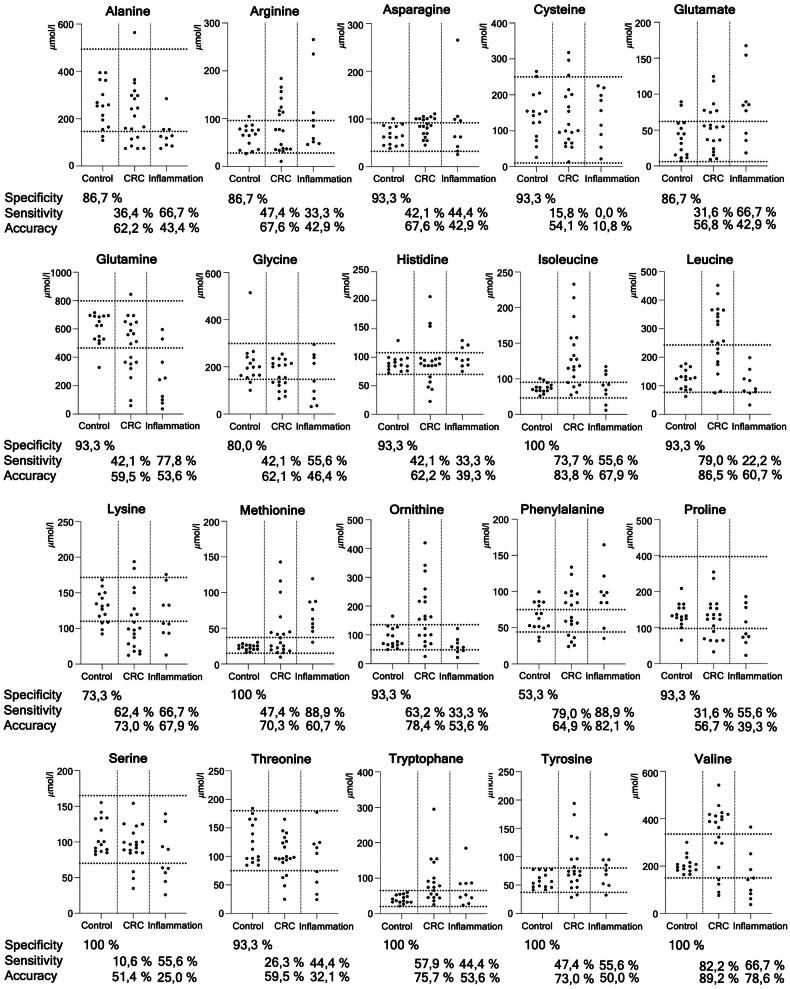
Individual metabolite expression in urine samples in control persons, colon adenocarcinoma patients (CRC), and patients diagnosed with different inflammations. Each dot corresponds to a person. The space between the hatched lines shows the normal range of amino acid expression. Specificity refers to the percentage of control persons that were correctly identified not to have metabolic anomalies. Sensitivity refers to the percentage of cancer (CRC) and inflammation patients that were correctly identified to have an anomaly in metabolism. Accuracy refers to the percentage of all persons that were correctly identified either not to have a disease (control persons) or to have a disease (cancer and inflammation patients).

The analysis of amino acid profiles in urine samples revealed that each patient exhibited a distinct pattern of metabolite alterations, complicating the direct interpretation of results. While urinary amino acids secretion including isoleucine, leucine, and valine, in cancer patients demonstrated substantial interindividual variability, amino acid secretion in control individuals remained predominantly withing normal ranges (Fig. 5). Additionally, specific subsets, such as ornithine and phenylalanine, displayed wide variability, with some patients exhibiting abnormally high and others abnormally low expression levels.

Specificity analysis, defined as the ability to correctly identify control individuals without the disease, ranged from 53.3% (phenylalanine) to 100% (isoleucine, methionine, serine, tryptophan, tyrosine, and valine). Sensitivity, reflecting the test’s ability to correctly identify individuals who have the disease, varied significantly. The lowest sensitivity values were observed for cysteine (15.8%), serine (10.6%) and threonine (26.3%), while a subset of amino acids, including alanine, arginine, asparagine, glutamate, glutamine, glycine, histidine, methionine, and proline, demonstrated sensitivities ranging from 31.6% to 47.4%. The highest sensitivity levels were observed for isoleucine (73.7%), leucine (79.0%), phenylalanine (79.0%), and valine (82.2%). Accuracy, representing the overall correctness of classification for both diseased and healthy individuals, was highest for isoleucine (83.8%), leucine (86.5%), and valine (89.2%) (Fig. 5).

The calculation of combined sensitivity based on the expression anomalies of isoleucine, leucine, phenylalanine, and valine yielded a sensitivity of 47.7% for isoleucine alone, 73.7% for isoleucine and leucine, 94.7% for isoleucine, leucine, and phenylalanine, and 100% for the combination of isoleucine, leucine, phenylalanine, and valine (Supplemental Table T1).

### Discrimination of cancer from inflammation

Although metabolic abnormalities are shared between cancer and inflammatory conditions, potentially leading to false-positive results [24–26], sensitivity analysis of glutamine, leucine, methionine, and ornithine suggests that these metabolites may facilitate the differentiation of malignancy from inflammation. Specifically, the sensitivity of individual metabolite values was 42.1% for glutamine in adenocarcinoma and 77.8% in inflammation; 31.6% for glutamate in adenocarcinoma versus 66.7% in inflammation; 79.0% for leucine in adenocarcinoma versus 22.2% in inflammation; 47.4% for methionine in adenocarcinoma compared to 88.9% in inflammation; and 63.2% for ornithine in adenocarcinoma relative to 33.3% in inflammation (Fig. 5).

When analyzing the combined biomarker values of glutamate and glutamine, the specificity, sensitivity, and accuracy in the cancer cohort were 90%, 39%, and 62%, respectively, whereas in the inflammation cohort these values were 90%, 72%, and 83%. Similarly, combined analysis of leucine and ornithine yielded specificity, sensitivity, and accuracy values of 93%, 71%, and 97% for cancer, compared to 93%, 27%, and 69% for inflammation. These findings indicate that while glutamate and glutamine exhibit lower sensitivity for cancer relative to inflammation, leucine and ornithine display higher sensitivity in cancer than in inflammatory conditions (Table 1).

**Table 1:** The combined analysis of glutamate and glutamine, as well as leucine and ornithine, in cancer and inflammation cohorts reveal distinct sensitivity patterns.

	Cancer	Inflammation
	**Glutamate and glutamine**	
Specificity	90%	90%
Sensitivity	39%	72%
Accuracy	62%	83%
	**Leucine and ornithine**	
Specificity	93%	93%
Sensitivity	71%	27%
Accuracy	97%	69%

The sensitivity of glutamate and glutamine for cancer diagnosis was 39%, which was lower than that observed for inflammation (72%). In contrast, the combined analysis of leucine and ornithine demonstrated higher sensitivity for cancer (71%) compared to inflammation (27%).

**Fig. 6:**
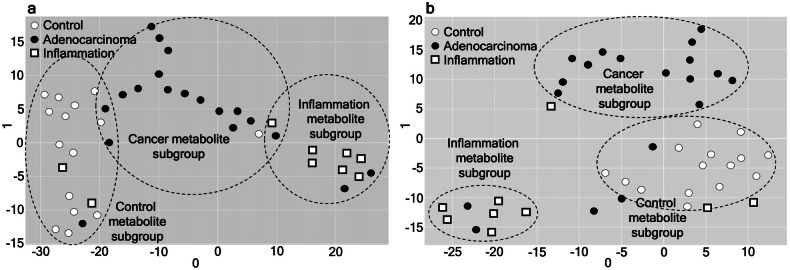
Visualization of metabolic profiles using t-SNE. The t-SNE analysis of metabolite expression patterns in urine samples from healthy controls, colon adenocarcinoma patients, and individuals with inflammation. Each symbol refers to an individual patient. **a** Analysis of glutamate, glutamine, leucine, and ornithine. The analysis shows a tight control cluster on the left side, a central cancer metabolic cluster that slightly overlaps with controls, and a distinct far right inflammation metabolic cluster. **b** Analysis of all 20 amino acids. The analysis shows a compact control metabolic cluster towards the bottom right, while the cancer cluster is shifted upper right, and the inflammation cluster is located at the lower left corner.

To evaluate the potential of amino acid metabolites for cancer diagnosis, we employed t-SNE (t-Distributed Stochastic Neighbor Embedding) [27]. As illustrated in Fig. 6, the t-SNE analysis provided a clear separation between controls, colon cancer patients, and inflammation cases, reinforcing the previous observations stating that specific metabolic alterations are associated with cancer progression. Analysis of the four-amino-acid panel (glutamate, glutamine, leucine, and ornithine) and all biomarkers four amino acid panel (Fig. 6a), the analysis of all biomarkers (Fig. 6b) both identified distinct metabolic subgroups for controls, cancer, and inflammation highlighting their potential for cancer-specific biomarker discovery, though inflammatory biomarker classification may require further refinement.

## Discussion

While the effect of tumor stroma paracrine factors and secreted metabolites on cancer cell function remains incompletely understood, recent studies emphasize the significance of reciprocal metabolic reprogramming between cancer cells and TAFs. Characteristically, tumor stroma TAFs favor anaerobic metabolism producing metabolic byproducts that cancer cells utilize, whereas cancer cells can preferentially engage in aerobic metabolism during these interactions, indicating the division of growth-promoting roles in tumorigenesis [22, 24–26, 28, 29].

In our study, we observed a marked variation in vitro metabolite secretion from TAFs isolated from adenocarcinomas (Fig. 4) Although the in vitro data indicated a significant trend in the transition from non-metastasized to metastatic cancer, the patient data did not support the observation. Notwithstanding, the mechanism behind the phenomena remains elusive, the rapid tumor expansion and aberrant neovascularization, in general, lead to nutrient deprivation. The subsequent metabolic adaptation to low nutrient levels triggers metabolic plasticity in cancer cells, characterized by alterations in glucose and glutamine metabolism [30]. We identified significant in vitro variations in the production of biomarkers, such as amino acids, glyceraldehyde, lactic acid, pyroglutamic acid, and succinic acid in fibroblasts isolated from non-metastasized and metastasized cancer. These metabolites serve diverse functions within cells: they can act as intermediates in amino acid synthesis, contribute to energy production, facilitate cellular growth as oncometabolites, and even play roles in regulating innate immunity [31]. The metabolites in question are synthesized from intermediates of the glycolytic pathway, the citric acid cycle, and the pentose phosphate pathway.

Most notably, the inconsistency in the metabolic expression in patient urine samples possesses diagnostic value that could reinforce the existing biomarker panels for detecting metabolic reprogramming indicative of tumorigenesis or inflammation [32, 33]. Persistent inflammatory signals can promote tumorigenesis by altering cellular metabolism and modulating an immunosuppressive microenvironment by creating pro-tumorigenic niches that facilitate cancer cell proliferation and survival [20, 26, 34].

Although the patient cohorts of the current study are small, the comparative sensitivity analysis of metabolic profiles between inflammatory and malignant states could facilitate the differentiation of these pathologies improving the specificity of biomarker-based cancer diagnostics (Fig. 5, Table 1). This was further supported by the t-SNE analysis, which by preserving local similarities and minimizing global distortions separated cancer metabolic subgroup from inflammation metabolic subgroup (Fig. 6). Nevertheless, our results suggest that urine metabolites could be used for cancer diagnosis. The use of the t-SNE nonlinear dimensionality reduction technique for data visualization accurately distinguishes inflammatory responses from malignant metabolic changes. The observed increases and decreases in amino acid levels suggest corresponding activation and inhibition of oxidative phosphorylation, glycolysis, the pentose phosphate pathway, and citric acid cycle, although the roles of these oncometabolites in tumor stroma or cancer cells remains unclear and warrant further investigation.

## Material and methods

### Cell lines and patient-derived primary cells

Cell lines, CaCo_2_ [35], DLD1, and HCT116 [36], were cultured in the conditions recommended by the distributor. Characteristics of the cells are described by “*Cellosaurus, Amos Bairoch, CALIPHO group, SIB - Swiss Institute of Bioinformatics, Lausanne Switzerland*” (Fig. 1). Primary cells at passage 6–8 were used in the study. Diagnosis of primary cell donor patients; Adenoma patient: Tubulovillous adenoma with low-grade dysplasia. Adenocarcinoma patient: Moderately differentiated intestinal adenocarcinoma infiltrating the full thickness of the muscular wall and extending to the subserosa. Reactive lymph nodes. Chronic cholecystitis. Pathologic stage: pT3 N0 MX. Metastasized adenocarcinoma patient: Moderately differentiated adenocarcinoma with early infiltration of adipose tissue. Metastatic involvement of one lymph node. Pathologic stage: pT3 N1, Dukes’ stage C2.

### BrdU DNA replication analysis

To assess cell proliferation, bromodeoxyuridine (BrdU) (Roche, Basel, Switzerland). BrdU visualization and counterstaining of nuclei were done with Hoechst dye (Sigma, St. Louis, MO, USA).

### Matrigel migration analysis

Cancer cell migration towards primary cultures was done using Matrigel (Corning, Corning, NY, USA) and migration chambers (8 microns, BD, San Jose, CA, USA). Migrated cancer cells were visualized with crystal violet (Sigma).

### Gene expression analysis

Isolated mRNA (RNeasy Mini Kit, Qiagen, Hilden, Germany) was reverse transcribed to cDNA (QuantiTect Reverse Transcription Kit, Qiagen). qPCR was performed using SYBR Green PCR Master Mix (Applied Biosystems, Foster City, CA, USA). Primers are listed in the supplemental file of Materials and Methods.

### Analysis of secreted proteins and cytokines in colon naïve fibroblasts and colon adenocarcinoma TAFs

R&D Systems array (Minneapolis, MI, USA) was employed to assess the expression of growth factors from whole-cell lysates (70 µg).

### Western blot analysis and small GTPase RAS and RAC pulldown analysis

Protein lysates were probed with antibodies pERK1/2, ERK1/2, pAKT T308, pAKT S473, AKT, pp38MAPK, p38MAPK, Tubulin, pJNK, JNK, pSMAD, pGSK3β, β Catenin, PKA substrates, pCREB, CREB, and actin. All antibodies are from Cell Signaling Technologies, Danvers, MA, USA. The supernatant from lysed cells was incubated with GST-Raf1-RBD beads. Active Ras G-protein-containing beads were collected, run in gel, and probed by mouse anti-Ras and anti-RAC (Upstate, Charlottesville, VA, USA).

### Seahorse XF analyzer respiratory assay

Cellular oxygen consumption rate (OCR) and extracellular acidification rate (ECAR) measurements were conducted using the XF96 Extracellular Flux Analyzer (Seahorse Bioscience, Houston, TX, USA) in conjunction with the XF Cell Mito Stress Test (Agilent, Santa Clara, CA, USA).

### Oncometabolite detection from fibroblast culture medium

The detection was done using gas chromatography-mass spectrometry (Agilent Technologies, Palo Alto, CA, USA).

### Urine analysis of patient samples

Total of 15 persons were selected as controls. The cancer patient cohort contained 19 patients. Nine patients diagnosed with inflammation were used to address incidence of false positives. Urine samples were analyzed with LCMS 8050 (Shimadzu, Kyoto, Japan) using amino acid detection kit and amino acid standards mixture (Supelco, Sigma).

### t-Distributed Stochastic Neighbor Embedding (t-SNE) analysis of the metabolite expression data

The analysis was conducted in Python using the scikit-learn library with the following parameter settings: Perplexity: 30, Learning rate: 200, Number of iterations: 1000, Metric: Euclidean distance. Following the t-SNE projection, sample clusters were analyzed to determine metabolic similarities and differences between groups.

### Ethical statement

Ethical approvals for the isolation of the primary cells and urine collection were obtained from the ethical committees of the Monaldi Hospital, Naples, Italy (Project 2013-01-02, deliberation number 1293), the University of Federico II of Naples, Naples, Italy (Protocol 394/19), and from the Clinical Mediterranea hospital, Naples, Italy (Authorization released by Medical Director on 24.5.2023). Informed consent was obtained from all participating patients.

### Statistical analysis

For the statistical validation of t-SNE analysis, we conducted: 1) Silhouette Score, 2) Principal Component Analysis (PCA), and 3) Machine Learning Classifiers. Statistical analysis of cell proliferation, migration, and protein activation was conducted using a two-tailed independent samples *t*-test. For the analysis of Seahorse data, ANOVA followed by Bonferroni post hoc test was utilized. Results are expressed as the mean standard deviation (SD). The p-values are reported as follows: *p < 0.05, **p < 0.01, ***p < 0.001.

Full description of the methods is described in the supplemental Materials and Methods file.

## Supplementary information

Supplemetary file texts

Supplementary files

Materials and Methods

Original Western blots

## Data Availability

All data are available in the main text or the supplementary materials.
