# Supplementary material for: Oncometabolite signatures from tumor-stroma crosstalk as potential non-invasive biomarkers
Source: Cell Death Discov. 2026 May 22;12:306. doi: 10.1038/s41420-026-03172-1 (PMC13373187; doi:10.1038/s41420-026-03172-1)
Supplement: Supplementary file 1 — Supplemetary file texts [file 41420_2026_3172_MOESM1_ESM.docx]

**Supplementary Fig. S1. Screening of stromal factors**. Naïve fibroblast and TAF screening from non-metastasized and metastasized colon adenocarcinoma showed minor changes in common and non-hematopoietic marker expression.

**Supplementary Fig. S2. Signal transduction analysis of fibroblasts and patient’s colon tissue.** **a.** The phosphorylation levels of ERK1/2, AKT (T308 and S473), and p38MAPK were at similar levels in naïve fibroblasts and TAF isolated from adenoma, non-metastasized, and metastasized colon cancer. **b, c**. Small GTPase RAS and RAC pulldown analysis of HCT116 cells co-cultured with fibroblasts suggested a similar kind of fibroblast paracrine effect on cancer cell signaling. **d.** Signaling analysis from colon tissue homogenates. The activation of signal transduction from normal colon, adenoma, and adenocarcinoma tissues both from non-metastasized and metastasized cancers showed moderate changes.

**Supplementary Table T1**

**Urine analysis of amino acid expression**. The analysis of 15 control persons, 19 colon cancer patients, and 9 inflammation patients validated *in vitro* anomalies in oncometabolite expression in patients (squared values).
