# Supplementary material for: Oncometabolite signatures from tumor-stroma crosstalk as potential non-invasive biomarkers
Source: Cell Death Discov. 2026 May 22;12:306. doi: 10.1038/s41420-026-03172-1 (PMC13373187; doi:10.1038/s41420-026-03172-1)
Supplement: Supplementary file 2 — Supplementary files [file 41420_2026_3172_MOESM2_ESM.pdf]

Supplementary Fig. S1

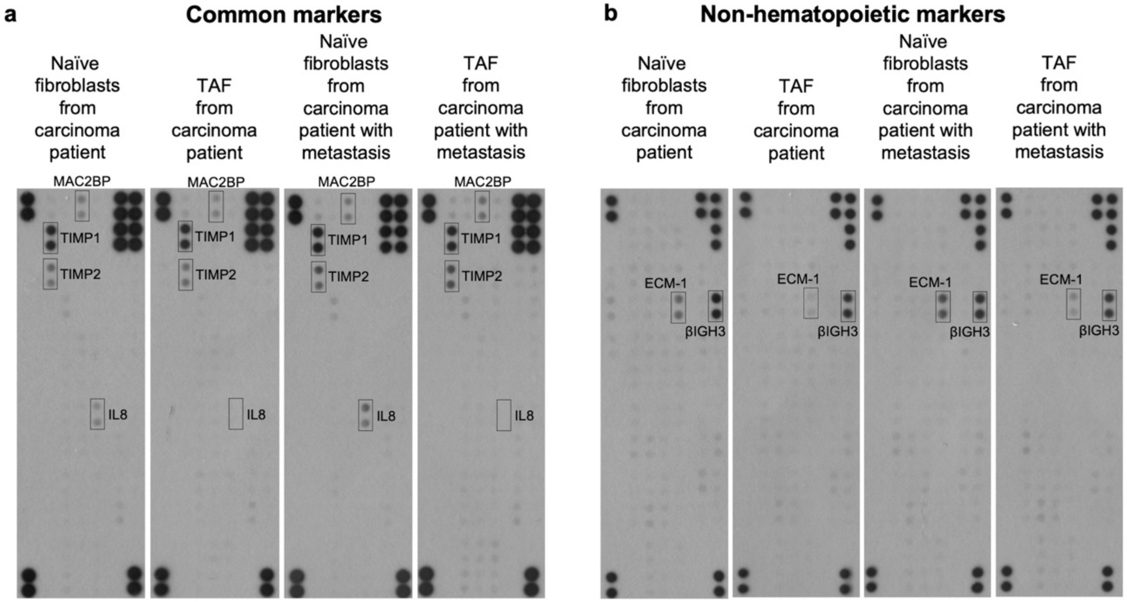

Supplementary Fig. S2.

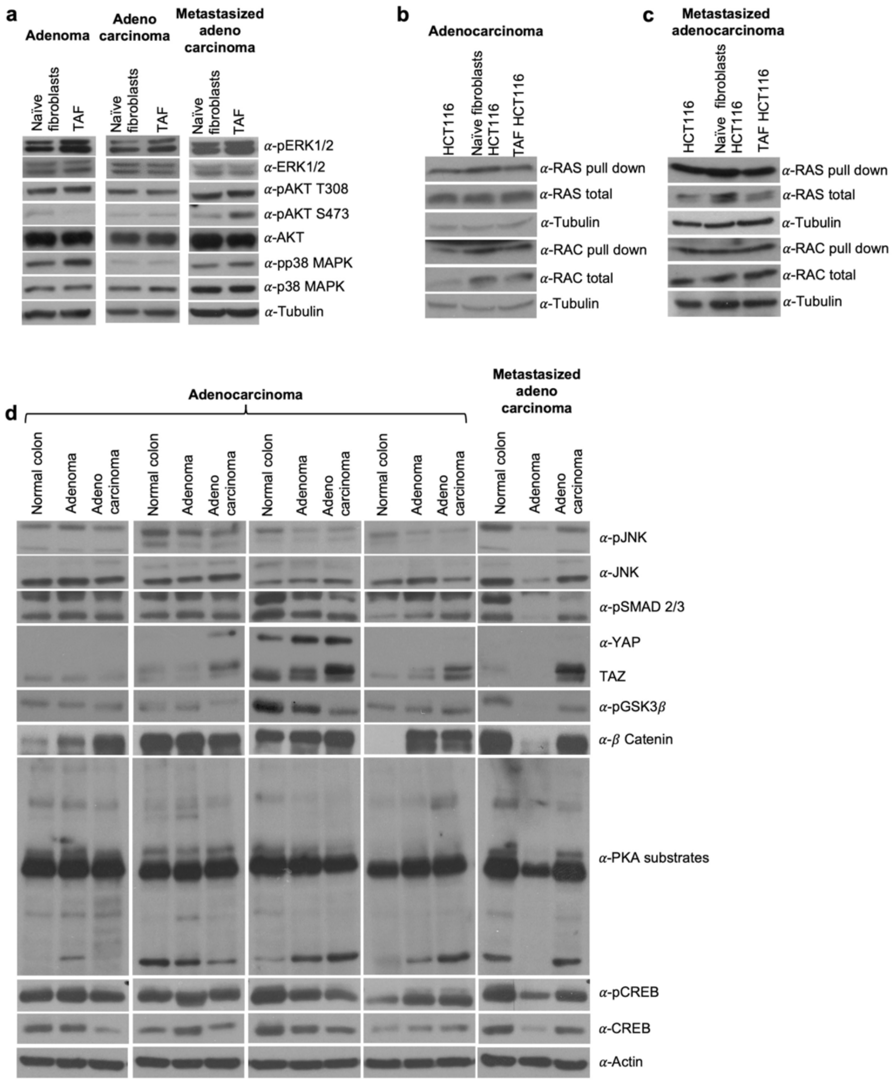

Supplemental Table T1

CONTROL PERSONS

Normalvalue ra	146-494	28-96	32-92	10-250	6-62	466-798	147-299	70-108	46-90	77-205	120-243	15-37	48-135	44-75	97-297	70-165	75-180	20-65	37-80	150-335
	Alanine	Arginine	Asparagine	Cysteine	Glutamate	Glutamine	Glycine	Histidine	Isoleucine	Leucine	Lysine	Methionine	Ornithine	Phenylalanine	Proline	Serine	Threonine	Tryptophane	Tyrosine	Valine
Control 1	254,2	67,7	84,6	122,3	15,9	624,8	163,7	72,6	75,4	154,2	117,5	26,2	130,8	85,6	127,3	91,4	89,5	59,7	63,2	188,3
Control 2	394,7	75,6	61,7	147,2	7,2	328,7	257,3	91,3	51,8	168,7	218,7	20,7	127,3	52,6	64,7	86,7	154,7	32,9	77,5	207,3
Control 3	154,8	84,5	89,4	204,8	11,4	528,9	134,9	78,4	65,2	89,6	200,6	26,1	121,4	36,9	109,5	132,6	96,3	32,6	77,4	201,4
Control 4	108,5	78,4	42,8	265,4	52,4	695,8	200,4	99,6	89,3	132,9	96,5	16,8	96,5	31,8	154,2	155,2	165,4	36,5	41,9	198,4
Control 5	301,8	104,5	46,9	142,1	84,5	691	265,4	96,4	96,4	177,4	139,4	22,1	165,4	99,6	124,5	133,8	99,4	55,8	48,7	300,4
Control 6	362,4	31,2	38,4	84,5	38,4	517,8	198,5	76,2	82,1	166,9	154,2	16,2	65,9	52,9	129,4	96,3	84,6	54,8	77,4	207,4
Control 7	125,4	48,5	61,4	72,9	89,4	652,9	100,5	96,3	66,2	96,3	169,2	20,4	69,8	51,7	132,4	84,9	84,5	41,2	47,2	184,3
Control 8	254,1	26,5	100,7	154,2	44,8	622,8	230	84,5	76,3	85,3	84,6	25,4	99,6	69,8	136,2	141,8	96,5	52,8	41,8	164,2
Control 9	268,9	35,4	64,8	154,9	22,6	689,4	194,6	84,2	56,8	132	165,4	21,5	51,9	69,4	165,2	82,4	93,4	45,4	46,9	254,8
Control 10	394,5	86,5	62,4	200,3	45,1	684,9	241,5	86,4	73,2	125,4	196,4	26,5	76,4	51,4	121,3	100,6	126,4	32,6	67,4	194,8
Control 11	214,9	76,4	45,1	123,9	31,6	529,4	165,2	75,4	66,3	86,9	134,6	23,6	64,9	49,5	132,6	86,9	165,4	26,9	53,4	182,4
Control 12	194,8	65,3	44,5	154,2	11,5	694,5	215,4	84,5	66,5	121,4	115,4	28,4	69,8	62,8	165,3	89,4	112,5	32,4	45,9	236,5
Control 13	365,2	33,6	86,4	25,9	59,6	497,5	165,2	89,5	76,4	125,4	184,9	25,4	69,8	86,2	208,4	133,6	175,4	21,5	56,9	164,8
Control 14	164,9	74,9	69,8	55,4	60,2	547,9	514,2	129,4	69,8	62,9	162,4	30,4	59,4	80,2	99,5	100,4	139,4	48,5	56,4	179,4
Control 15	258,8	64,5	82,4	251,4	15,4	714,54	154,9	98,6	100,5	128,4	236,1	20,5	48,9	85,4	154,2	116,5	184,2	35,9	76,4	215,4

COLON CANCER PATIENTS

Normalvalue ra	146-494	28-96	32-92	10-250	6-62	466-798	147-299	70-108	46-90	77-205	120-243	15-37	48-135	44-75	97-297	70-165	75-180	20-65	37-80	150-335
	Alanine	Arginine	Asparagine	Cysteine	Glutamate	Glutamine	Glycine	Histidine	Isoleucine	Leucine	Lysine	Methionine	Ornithine	Phenylalanine	Proline	Serine	Threonine	Tryptophane	Tyrosine	Valine
Patient 1	284,1	156,7	69,7	65,7	9,1	321,4	214,3	85,4	274,9	368,7	74,5	29,7	259,4	123,7	63,7	84,4	165,2	74	96,3	425,7
Patient 2	74,7	36,1	84,5	194,7	19,8	694,3	134,7	93,7	327,7	422,9	287,4	19,7	341,7	25,7	68,4	124,9	96,8	44,5	174,3	396,1
Patient 3	297,5	77,3	89,5	317,7	36,7	844,5	132,7	96,7	365,8	451,9	117,6	9,8	419,7	96,7	152,9	95,6	144,8	294,7	194	362,7
Patient 4	159,7	184,2	100,6	117,8	15,2	632,8	200,4	98,3	214,8	352,4	139,6	20,5	162,9	133,8	64,5	100	62,8	154,2	136,2	411,7
Patient 5	74,2	31,8	84,5	54,1	36,2	352,9	206,4	98,4	124,9	214,8	36,2	25,4	165,2	64,2	122,8	91,5	133,2	78,4	74,5	298,6
Patient 6	84,5	10,5	105,4	166,5	74,8	651,2	74,6	85,4	154,2	256,7	36,5	25,4	98,4	36,2	135,2	122,4	123,7	91,4	133,4	418,7
Patient 7	351,2	124,1	104,5	154,2	24,1	365,2	154,7	56,8	165,5	254,9	56,8	22,7	231,4	84,5	154,2	88,4	99,6	145,1	74,5	385,4
Patient 8	298,5	74,5	45,2	200,7	77,5	399,4	232,1	88,5	215,4	365,9	98,4	16,2	154,2	94,5	236,4	112,4	98,4	100,4	45,1	541,8
Patient 9	121,5	33,6	100,5	56,2	52,4	566,4	236,4	94,6	133,7	341,8	24,9	41,5	321,7	98,4	165,7	125,4	89,4	45,4	95,6	455,8
Patient 10	564,1	35,4	100,4	296,4	55,9	587,9	136,4	159,4	77,5	141,5	155,2	44,9	214,8	54,8	135,9	99,6	48,5	25,4	69,5	142,8
Patient 11	114,2	45,8	110,8	254,8	56,9	695,3	234,1	47,8	136,3	326,4	28,4	65,9	25,4	84,5	254,1	88,9	66,5	54,2	55,9	418,4
Patient 12	156,9	36,5	54,8	99,4	54,2	256,9	155,9	88,9	154,8	249,6	49,9	15,9	216,4	56,4	89	48,7	95,6	65,2	58,3	388,4
Patient 13	365,2	108,4	69,5	100	86,5	495,2	254,1	65,8	129,5	365,2	200,4	36,5	121,9	59,4	135,9	96,5	141,2	88,5	74,5	408,9
Patient 14	165,4	77,2	81,1	95,6	35,2	559,6	214,9	85,4	195,4	314,8	95,6	44,1	100,2	100,4	165,2	84,5	96,3	36,2	46,2	322,8
Patient 15	211,9	142,5	100,5	65,8	54,8	647,5	208,4	86	105,4	184,5	100,9	41,8	98,5	39,6	125	99,6	126,3	45,8	66,9	296,4
Patient 16	318,4	37,8	61,7	13	10,2	511,2	121,5	43,8	61,7	80,9	137,8	18,2	60,9	30,2	104,5	58,3	93,8	74,5	67,6	123,5
Patient 17	241,8	113,6	54,6	95,4	124,6	58,4	96,3	22,5	54,6	75,4	214,4	100,5	154,6	24	62,8	85,4	96,3	55,4	32,9	89,5
Patient 18	245,1	165,9	86,4	76,8	76,9	365,2	95,4	206,4	89,6	174,3	268,4	142,9	69,5	81,4	122,5	34,9	24,8	154,8	82,4	76,2
Patient 19	74,4	116,5	98,4	214,6	118,4	94,5	65,4	154,2	82,4	229,5	84,5	116,2	76,4	58,9	32,6	154,2	116,3	76,4	28,4	194,5

INFLAMMATION PATIENTS

Normalvalue ra	146-494	28-96	32-92	10-250	6-62	466-798	147-299	70-108	46-90	77-205	120-243	15-37	48-135	44-75	97-297	70-165	75-180	20-65	37-80	150-335
	Alanine	Arginine	Asparagine	Cysteine	Glutamate	Glutamine	Glycine	Histidine	Isoleucine	Leucine	Lysine	Methionine	Ornithine	Phenylalanine	Proline	Serine	Threonine	Tryptophane	Tyrosine	Valine
Inflammation 1	128	51,7	62,8	156,3	18,2	529,7	139,8	97,7	56,7	81,9	88,3	30,4	121,9	84,5	168,9	128,7	105,3	23,7	68,7	98,7
Inflammation 2	74,1	47,8	105,6	198,4	45,8	365,2	295,4	96,2	122,4	198,6	165,2	51,2	56,9	84,5	155,9	89,5	177,3	86,5	139,4	364,8
Inflammation 3	118,4	84,5	62,3	219,4	85,4	596,4	239,4	121,5	68,4	89,5	251,4	45,7	55,8	164,5	185,9	44,5	115,4	85,4	94,6	185,2
Inflammation 4	154,9	58,4	99,4	184,6	35,6	241,9	96,8	75,4	82,6	32,9	112,4	56,2	41,5	49,2	72,8	63,8	33,6	28,4	49,5	251,8
Inflammation 5	84,5	265,3	25,4	225,1	154,3	100,4	35,6	94,5	25,7	124,3	114,2	87,6	21,7	98,6	74,5	25,9	24,3	84,5	76,2	62,4
Inflammation 6	125,4	45,9	96,4	54,2	76,9	124,63	214,6	86,5	134,2	118,4	86,4	76,4	83,5	121,4	82,5	93,4	121,7	52,9	32,4	149,7
Inflammation 7	154,9	235,1	42,1	89,6	167,41	77,2	31,4	129,4	112,5	74,9	25,4	86,9	71,4	35,2	22,9	56,9	124,5	184,7	94,5	142,9
Inflammation 8	89,4	112,4	265,4	21,4	89,4	254,9	65,3	84,9	11,4	78,9	165,2	62,9	45,8	99,6	115,4	139,4	54,8	46,63	85,4	37,5
Inflammation 9	284,5	96,4	32,6	115,4	84,5	35,9	251,4	116,9	84,2	157,9	235,4	119,4	58,7	94,6	58,3	62,9	73,5	44,9	52,4	83,1
