# Supplementary material for: Oncometabolite signatures from tumor-stroma crosstalk as potential non-invasive biomarkers
Source: Cell Death Discov. 2026 May 22;12:306. doi: 10.1038/s41420-026-03172-1 (PMC13373187; doi:10.1038/s41420-026-03172-1)
Supplement: Supplementary file 3 — Materials and Methods [file 41420_2026_3172_MOESM3_ESM.docx]

**MATERIALS AND METHODS**

**Study design**

The aim of this work was to study the contribution of stromal cells to the tumor microenvironment and their interactions with cancer cells, to identify the stromal cells secreted mediators promoting carcinogenesis, and to assess the diagnostic validity of the identified mediators in colon adenocarcinoma. For in vitro studies, we selected colon fibroblasts from non-metastasized cancer that supported either proliferation of migration of colon cancer cells, respectively. The aim of the *in vitro* studies was to identify stromal paracrine factors that mediate the tumor stroma interaction. The aim of the patient studies was to validate the abnormalities in the expression of the stromal mediators from urine samples. Due to low numbers of persons included into each cohort, we selected homogenous populations of persons to minimize the interpersonal differences in the expression of the stromal mediators.

**Cell lines and patient-derived primary cells**

All primary cells were cultured in αMEM (Euroclone, Milano, IT) supplemented with 10 % defined fetal bovine serum (FBS) (GE-Healthcare, Chicago, IL, USA), non-essential amino acids (Euroclone) L-alanine-L-glutamine (Euroclone), and penicillin/streptomycin (Euroclone). Colorectal carcinoma CaCo2, DLD1, and HCT116 cells (ATTC, Manassas, VA, USA) were grown in DMEM (Euroclone) supplemented with 10 % FBS (Euroclone), glutamine (Euroclone) and penicillin/streptomycin (Euroclone).

Ethical approval for the isolation of colon fibroblasts and urine collection was obtained from the ethical committees of Monaldi Hospital, Naples, Italy (Project 2013-01-02, deliberation number 1293), the University of Naples Federico II, Naples, Italy (Protocol 394/19), and from the Clinical Mediterranea hospital, Naples, Italy (Authorization released by Medical Director on 24.5.2023). Informed consent was obtained from all participating patients. The study was performed in accordance with the Declaration of Helsinki.

Diagnosis of primary cell donor patients; **Adenoma patient**: Tubulovillous adenoma with low-grade dysplasia. **Adenocarcinoma patient**: Moderately differentiated intestinal adenocarcinoma infiltrating the full thickness of the muscular wall and extending to the subserosa. Reactive lymph nodes. Chronic cholecystitis. Pathologic stage: pT3 N0 MX. **Metastasized adenocarcinoma patient**: Moderately differentiated adenocarcinoma with early infiltration of adipose tissue. Metastatic involvement of one lymph node. Pathologic stage: pT3 N1, Dukes’ stage C2.

Primary benign adeno TAFs, adenocarcinoma TAFs, and their naïve counterpart fibroblasts (isolated approximately 20 cm from the tumor from a normal segment of the colon that was histologically confirmed to be free of neoplasia) were isolated by cutting the tissue into small pieces, which were then incubated overnight at 37 °C in αMEM (Euroclone) supplemented with antibiotic-antimycotic and gentamicin, along with collagenase type II at 150 U/ml (Gibco, Waltham, MA, USA). The following day, undigested tissue fragments were removed via centrifugation, and the supernatant was plated onto gelatin (1 g/l) coated dishes in αMEM supplemented with 10 % defined fetal bovine serum, non-essential amino acids, L-alanine-L-glutamine, penicillin/streptomycin, antibiotic-antimycotic and gentamicin. The cell culture medium and antibiotics were refreshed daily during the initial weeks of the primary cell isolation. The cells were passaged for the first time when the culture reached approximately 30 % confluence. Primary fibroblasts at passages 6-8 were utilized for the study.

**BrdU DNA replication analysis**

To investigate the impact of primary mesenchymal cell on cancer cell proliferation, CaCo_2_, DLD1, and HCT116 cells were initially seeded onto coverslips (Waldemar Knittel Glasbearbeituns-GmbH, Braunschweig, Germany) and allowed to grow until reaching 60-70 % confluence. Following this, the coverslips were incubated overnight in the presence of primary cell cultures. Naïve colon fibroblasts and colon TAFs, which were approximately 70-80 % confluent at the time of co-culture, were prepared three days in advance in 100x200 mm dishes to facilitate the concentration of paracrine factors in the cell culture medium.

To assess cell proliferation, bromodeoxyuridine (BrdU) was added to the co-culture medium at a concentration of 10 mM (Roche, Basel, Switzerland) and allowed to incubate for 15 min. The HCT116 cells grown on the coverslips were subsequently fixed using an ethanol fixative solution. BrdU-positive cells were then detected using FITC-conjugated secondary antibodies (Jackson Immuno Research Laboratories Inc. West Grove, PA, USA). Nuclei were counterstained with Hoechst dye (Sigma, St. Louis, MO, USA) to enable visualization of cell morphology. BrdU-positive cells were counted across high-power fields using a microscope. As controls, naïve counterpart fibroblasts isolated from the same patients as the TAFs were employed. The experiments were repeated at least three times.

**Matrigel migration analysis**

For the chemotactic invasion assay, 100 μl of Matrigel (Corning, Corning, NY, USA) at a concentration of 1 mg/ml was added to migration chambers (8 microns, BD, San Jose, CA, USA) and allowed to stabilize at room temperature for 30 minutes. To evaluate the impact of primary cells on cancer cell migration, stromal cells were cultured in 12-well plates until reaching full confluence over a three-day period, enabling the concentration of paracrine factors in the medium. The experiments were repeated at least three times.

Subsequently, chambers containing 50 000 CaCo_2_, DLD1, or HCT116 cells embedded in Matrigel were transferred to the 12-well stromal cell cultures for an overnight incubation at 37 °C. The following day, Matrigel was carefully removed from the chambers, and the migrated cells were fixed with 7 % paraformaldehyde (Sigma). After fixation, the cells were washed with phosphate-buffered saline (PBS) and stained with crystal violet (Sigma) for visualization. The number of migrated cells was quantified in high-power microscope fields. As controls, naïve counterpart fibroblasts isolated from the same patient as the TAFs were utilized.

**Gene expression analysis**

For expression analysis, mRNA was isolated from cells using the RNeasy Mini Kit (Qiagen, Hilden, Germany) and subsequently reverse transcribed to cDNA using the QuantiTect Reverse Transcription Kit (Qiagen). Quantitative PCR (qPCR) was performed using SYBR Green PCR Master Mix (Applied Biosystems, Foster City, CA, USA). The following primers were utilized for gene amplification:

*FIBROBLAST GROWTH FACTOR 1* (*FGF1*) forward ctgcagtagcctggaggttc and reverse ggctgtgaaggtggtgattt, human *FGF2* forward ggtgaaaccccgtctctaca and reverse tctgttgcctaggctggact, human *FGF22* forward caggacagcatcctggagat and reverse gtaggtgttgtggccgttct, human *VASCULAR ENDOTHELIAL GROWTH FACTOR A* (*VEGF A*) forward cccactgaggagtccaacat and reverse tttcttgcgctttcgttttt, human *PLATELET DERIVED GROWTH FACTOR* (*PDGF*) forward gtggaggaaattgtggctgt and reverse, human *BONE MOPHOGENIC PROTEIN 5* (*BMP5*) forward gatgtgggttggcttgtctt and reverse acctcactcgccttgaagaa, human *BMP6* forward aagaaggctggctggaattt and reverse gaagggctgcttgtcgtaag, human *BMP7* forward tgtgatccctcttggtgtga and reverse caggttgaaggaaagcaagc, human *CCL2* forward gcagcaagtgtcccaaagaa and reverse ctggggaaagctaggggaaa, human *CCL4* forward tgcttttcttacaccgcgag and reverse cttcctgtctctgagcagct, human *CCL5* forward ccaaagagagagggacagca and reverse ctttcccagcctcactcaga, human *CCL19* forward tgatggtgggtgcctgtaat and reverse ggaaccctgagtaaagccct, human *CCL21* ggccttgacctctcctcttt and reverse gttctgaaaacctgccccag, human *CXCL11* forward cagttgttcaaggcttcccc and reverse gggtttaggcatcgttgtcc, human *CXCL12* forward ccggctgaagaacaacaaca and reverse ttttccttttctgggcagcc, human *IL2* forward cctcaactcctgccacaatg and reverse tgtgagcatcctggtgagtt, human *IL18* forward ctcagaccttccagatcgct and reverse gccgatttccttggtcaatga, human *IL21* forward aggtcaagatcgccacatga and reverse tgggccttctgaaaacagga, human *18SRNA* forward gttggttttcggaactgagg and reverse gcatcgtttatggtcggaac. The experiments were repeated at least three times.

**Analysis of secreted proteins and cytokines in colon naïve fibroblasts and colon adenocarcinoma TAFs**

A commercial array (R&D Systems, Minneapolis, MI, USA) was employed to assess the expression of growth factors in colon naïve fibroblasts and colon adenocarcinoma TAFs. Whole-cell lysates (70 μg) from the fibroblasts were utilized for analysis, following the manufacturer’s instructions. The proteins were visualized by enhanced chemiluminescence detection kit (ECL, GE Healthcare, Chicago, IL, USA). The experiments were repeated at least three times.

**Western blot analysis and Small GTPase RAS and RAC pulldown analysis**

For signaling analysis, cells were lysed using lysis buffer composed of 50 mM Hepes, pH 7.5, 150 mM NaCl, 10% glycerol, 1% Triton X-100, 1 mM EGTA, 1.5 mM MgCl2, 10 mM NaF, 10 mM sodium pyrophosphate, 1 mM Na3VO4, 10 μg of aprotinin/ml, 10 μg of leupeptin/ml (all from Sigma). Protein lysates were separated by SDS page and transferred on nitrocellulose membranes (GE Healthcare) that were probed with antibodies pERK1/2, ERK1/2, pAKT T308, pAKT S473, AKT, pp38MAPK, p38MAPK, Tubulin, pJNK, JNK, pSMAD, pGSK3β, β Catenin, PKA substrates, pCREB, CREB, and actin. All antibodies are from Cell Signaling Technologies, Danvers, MA, USA. After the antibody incubation, the signaling molecules were visualized by an enhanced chemiluminescence detection kit (ECL, GE Healthcare). The experiments were repeated at least three times.

Small GTPase RAS and RAC pull-down analysis was performed using cells at 40 % confluence in 10 cm dishes. After serum starvation for 24 h, the cells were lysed using ice-cold Rho-lysis buffer containing 20 mM HEPES (pH 7.4), 0.1 M NaCl, 1 % Triton X-100, 10 mM EGTA, 40 mM glycerophosphate, 20 mM MgCl2, 1 mM Na3VO4, 1 mM dithiothreitol, a mixture of protease inhibitors, and 1 mM phenylmethylsulfonyl fluoride (All reagents from Sigma). The lysates were incubated for 15 min with a purified, bacterially expressed GST-fusion protein containing the CRIB domain of PAK1 (p21 activated kinase) that had been previously bound to glutathione-Sepharose beads, followed by three washes using Rho-lysis buffer. GST-RAF1-RBD and GTP-bound forms of RAC1 associated with GST-CRIB beads were used for RAS and RAC pull-down assay, respectively, and quantified through Western blotting analysis. The experiments were repeated at least three times.

**Seahorse XF Analyzer respiratory assay**

Cellular oxygen consumption rate (OCR), which is an indictive of mitochondrial function, and extracellular acidification rate (ECAR), which indirectly indicates glycolytic activity, measurements were conducted using the XF96 Extracellular Flux Analyzer/Seahorse (Seahorse bioscience, Houston, TX, USA) in conjunction with the XF Cell Mito Stress Test (Agilent, Santa Clara, CA, USA). The energy metabolism of naïve fibroblasts, TAFs, and HCT116 cells was assessed by measuring both mitochondrial respiration and glycolysis with the Seahorse respirometer. To study the effect of tumor stroma on cancer cells, naïve fibroblasts and TAFs (1-2x10^4 cells) isolated from adenoma, and HCT116 (5000 cells) were seeded onto XFe96 culture miniplates for 24 h and allowed to stabilize for 48 h.

After stabilization of the cultures, conditioned medium collected after three-day culture from naïve fibroblasts and TAFs from adenoma, was added on HCT116 cells at time points 24 h, 48 h, and 62 h. The total culture time with conditioned medium was set to 76 h. Next, the cells were washed with EX base medium, supplemented with 10 mM glucose, 10 mM pyruvate, and 2 mM L-glutamine at pH 7.4, and subsequently moved to 37 °C non-CO_2_ incubator in 180 μl XF base medium. Similar protocol was applied to naïve fibroblast and TAF measurements.

Calibration measurements were taken under basal conditions and following the sequential injection of stressors: 1) Oligomycin, an inhibitor of ATP synthase, was administered at 1.5 μM concentration to naïve fibroblasts and TAFs, and at 1.0 μM concentration to HCT116 cells. 2) FCCP, an uncoupler that discharges mitochondrial membrane ΔpH potential was administered at 2.5 μM concentration to naïve fibroblasts and TAFs from adenomas and adenocarcinoma without metastasis, at 2.0 μM concentration to TAFs isolated from metastasized adenocarcinoma, and at 0.5 μM concentration to HCT116 cells. 3) Rotenone (0.5 μM) and antimycin A (0.5 μM), which inhibit complex I and complex III, respectively.

The concentrations of the stressors were optimized as follows: 1) OCR and ECAR values were normalized in pmol/min/10 000 cells. Basal OCR values were calculated by subtracting the endpoint after the addition of rotenone from the third measurement of the basal OCR value. Spare respiratory capacity, which reflects the potential ATP production by mitochondria under energy demand, was determined as the difference between the maximal OCR (achieved with FCCP) and the basal OCR. The experiments were repeated at least three times.

**Oncometabolite detection from fibroblast culture medium**

Chemicals and reagents

All chemicals and solvents (Carlo Erba, Milano, Italy) were analytical grade. N-tert-butyldimethylsilyl-N-methyl-trifluoroacetamide (MTBSTFA) and para-coumaric acid were purchased from Sigma.

Sample extraction and derivatization

Amino acids were analyzed by gas chromatography-mass spectrometry (GC-MS) after derivatization to their tert-butyldimethylsilyl (TBDMS) derivatives. Biological samples were treated with 100 μl of 0.1 M HCL:acetonitrile (1:1) solution. The mixtures were stirred for 2 min, sonicated for 1 min, and centrifuged for 2 min at 4000 rpm. A 50 μl aliquot was then evaporated to dryness under a gentle stream of nitrogen. Following evaporation, 50 μl of neat MTBSTFA and 500 μl of the internal standard solution (para-coumaric acid, 0.2 mg7ml in acetonitrile) were added. The mixture was heated at 80 °C for 1 h to complete the derivatization. The sample was then cooled and subjected directly to GC-MS analysis.

Gas chromatography-mass spectrometry

Analyses were carried out using GC-MS system consisting of an Agilent 6850A gas chromatograph and a 5973N quadrupole mass detector (Agilent Technologies, Palo Alto, CA, USA). Chromatographic separation was achieved using an Agilent HP5ms fused-silica capillary column (30 m x 0.25 mm, i.d. 0.25 µm film thickness, 5%-phenyl-95%-dimethylpolysiloxane). The injection was performed in spitless mode at 250 °C. The gas chromatography oven temperature was initially 80 °C for 1 min, then increased at 20 °C/min to 300 °C, and held for 15 min. Helium was the carrier gas, flowing at a constant rate of 1.0 ml/min. Mass spectra were recorded using electron impact ionization at 70 eV, with the ion source temperature set to 280 °C and a vacuum of 10⁻⁵ Torr. Mass spectral data were acquired simultaneously in TIC (m/z 50 to 600, 0.42 scans/s) and SIM modes. Gas Chromatography - Selected Ion Monitoring - Mass Spectrometry (GC-SIM-MS) analysis was performed selecting the following ions:

| **Compound** | **m/z** | **Compound** | **m/z** | **Compound** | **m/z** | **Compound** | **m/z** |
| --- | --- | --- | --- | --- | --- | --- | --- |
| Alanine | 158 | Glutamine | 431 | Lysine | 198 | Pyroglutamate | 300 |
| Asparagine | 417 | Glycine | 218 | Methionine | 218 | Serine | 288 |
| Aspartate | 302 | Histidine | 459 | N-Acetyl-Aspartate | 460 | Succinate | 289 |
| Coumaric acid (IS) | 335 | Hydroxyproline | 314 | Ornithine | 432 | Threonine | 303 |
| Cysteine | 406 | Isoleucine | 274 | Orotate | 441 | Tryptophan | 244 |
| Cystine | 348 | Lactate | 261 | Phenylalanine | 302 | Tyrosine | 302 |
| Glutamate | 432 | Leucine | 274 | Proline | 258 | Valine | 186 |

**Patients**

The exclusion criteria for patient selection included a body mass index (BMI) exceeding 24.9, the presence of metabolic syndrome, diabetes, recent cancer (with exception of ongoing colorectal cancer) ongoing pathologies that could affect metabolism (excluding inflammation), and the use of medications that influence metabolic processes. A total of 12 males and 3 females (n=15), aged between 45 and 60 years, were selected as controls. The cancer patient cohort contained 18 males and 1 female (n=19), aged 45 to 75 years, representing both non-metastasized and metastasized adenocarcinoma groups. To address incidence of false positives, we analyzed urine samples from 9 patients diagnosed with inflammation. Urine samples were collected in the morning. Prior to sample collection, patients were instructed to follow a low-carbohydrate diet rich in fiber and protein for three days and fast for 12 hours.

**Analysis of patient urine samples using liquid chromatography-mass spectrometry (LC-MS)**

Urine samples were analyzed at Centro Varelli, Naple, Italy, employing ISO9001:2005 certified liquid chromatography mass spectrometry, LCMS 8050 from Shimadzu, integrated with Shimadzu HPLC system (Kyoto, Japan). The amino acid standards utilized included a mixture from “Supelco” (Sigma) supplemented with pure standards of asparagine, glutamine, and ornithine. The chromatography separation was conducted using a Phenomenex C18 polar column (Sigma). For the mobile phases, Eluent A was prepared and contained 10 mM ammonium formate and 0.15 % formic acid, adjusted to a pH of 3.0. Eluent B was based on of acetynitrile and water solution in an 85:15 v/v ratio, 10 mM ammonium formate, and 0.15 % formic acid. The flow rate was set to 0.4 ml/min. Before analysis, the samples were treated with 0.1 % hydrochloric acid solution to achieve the desired ionic conditions, with pH adjusted to pH 5.0 (all reagents from Sigma). The injection volume for each sample was 5 μl.

The following outlines the specific parameters for the analysis:

Alanine: Precursor m/z 90.1, Product m/z 44, Dwell Time 3 msec, Collision Energy -14 V

Arginine: Precursor m/z 175, Product m/z 70, Dwell Time 3 msec, Collision Energy -19 V; Precursor m/z 175, Product m/z 60, Dwell Time 3 msec, Collision Energy -19 V

Asparagine: Precursor m/z 133, Product m/z 116, Dwell Time 3 msec, Collision Energy -13 V; Precursor m/z 133, Product m/z 74, Dwell Time 3 msec, Collision Energy -21 V

Aspartic Acid: Precursor m/z 134, Product m/z 74, Dwell Time 3 msec, Collision Energy -15 V

Cystine: Precursor m/z 122, Product m/z 76, Dwell Time 7 msec, Collision Energy -19 V

Glutamic Acid: Precursor m/z 148.1, Product m/z 84, Dwell Time 7 msec, Collision Energy -15 V

Glutamine: Precursor m/z 147.1, Product m/z 84, Dwell Time 7 msec, Collision Energy -24 V; Precursor m/z 147.1, Product m/z 130, Dwell Time 7 msec, Collision Energy -21 V

Glycine: Precursor m/z 76.1, Product m/z 76.1, Dwell Time 7 msec, Collision Energy -2 V

Histidine: Precursor m/z 156.1, Product m/z 110, Dwell Time 7 msec, Collision Energy -34 V

Isoleucine: Precursor m/z 132, Product m/z 86, Dwell Time 3 msec, Collision Energy -23 V; Precursor m/z 132, Product m/z 69, Dwell Time 3 msec, Collision Energy -51 V

Leucine: Precursor m/z 132.1, Product m/z 44, Dwell Time 3 msec, Collision Energy -31 V; Precursor m/z 132.1, Product m/z 43, Dwell Time 3 msec, Collision Energy -35 V

Lysine: Precursor m/z 147.1, Product m/z 130, Dwell Time 3 msec, Collision Energy -13 V; Precursor m/z 147.1, Product m/z 84, Dwell Time 3 msec, Collision Energy -30 V

Methionine: Precursor m/z 150.1, Product m/z 56, Dwell Time 2 msec, Collision Energy -22 V

Ornithine: Precursor m/z 133, Product m/z 70, Dwell Time 3 msec, Collision Energy -23 V

Phenylalanine: Precursor m/z 166.1, Product m/z 120, Dwell Time 3 msec, Collision Energy -35 V; Precursor m/z 166.1, Product m/z 77, Dwell Time 3 msec, Collision Energy -51 V

Proline: Precursor m/z 116.1, Product m/z 70, Dwell Time 3 msec, Collision Energy -23 V; Precursor m/z 116.1, Product m/z 43, Dwell Time 3 msec, Collision Energy -41 V

Serine: Precursor m/z 106, Product m/z 60, Dwell Time 3 msec, Collision Energy -23 V; Precursor m/z 106, Product m/z 42, Dwell Time 3 msec, Collision Energy -30 V

Threonine: Precursor m/z 120.1, Product m/z 103.2, Dwell Time 7 msec, Collision Energy -12 V

Tryptophan: Precursor m/z 205.1, Product m/z 188, Dwell Time 2 msec, Collision Energy -23 V; Precursor m/z 205.1, Product m/z 118, Dwell Time 2 msec, Collision Energy -36 V

Tyrosine: Precursor m/z 182.1, Product m/z 91, Dwell Time 2 msec, Collision Energy -38 V; Precursor m/z 182.1, Product m/z 136, Dwell Time 2 msec, Collision Energy -17 V

Valine: Precursor m/z 118.1, Product m/z 72, Dwell Time 3 msec, Collision Energy -38 V; Precursor m/z 118.1, Product m/z 55, Dwell Time 3 msec, Collision Energy -27 V

**t-Distributed Stochastic Neighbor Embedding (t-SNE) analysis of the metabolite expression data**

Data collection and preprocessing

Metabolic expression data were obtained from urine samples of three patient groups: healthy controls (n=15), colon cancer patients (n=9), and patients with inflammatory conditions (n=9). Data preprocessing included the following steps: Normalization: Metabolite concentrations were log-transformed to reduce skewness and ensure comparability across samples. Scaling: Data were standardized using z-score normalization, centering each metabolite’s distribution to have a mean of zero and unit variance. Missing values: Imputation was performed using the k-nearest (KNN) algorithm, preplacing missing values with estimates based on the most similar samples. Outlier detection: Extreme values were identified through interquartile range (IQR) analysis and excluded when necessary.

Dimensionality reduction using t-SNE

The analysis was conducted I Python using the scikit-learn library with the following parameter settings: Perplexity: 30 (optimized through multiple test runs). Learning rate: 200. Number of iterations: 1000 metric: Euclidean distance. The t-SNE algorithm computed pairwise similarities between samples in the high-dimensional space, then projected them into a two-dimensional (2D) embedding using a student’s t-distribution to minimize the Kullback-Leibler (KL) divergence.

Clustering and interpretation

Following the t-SNE projection, sample clusters were analyzed to determine metabolic similarities and differences between groups. The resulting 2D scatter plot revealed three distinct clusters corresponding to the control, cancer, and inflammation groups: Healthy controls formed a compact, well-defined cluster, indicating a relatively stable metabolic profile. Colon cancer patients exhibited a clearly separated grouping, reflecting significant metabolic alterations associated with malignancy. Inflammation patients displayed a more dispersed cluster, suggesting metabolic variability within the group. The metabolite distributions contributing to each cluster were further examined to identify potential biomarkers distinguishing cancer from inflammation.

**Statistical analysis**

For the statistical validation of t-SNE analysis, we conducted additional statistical analysis: 1) Silhouette score to assess clustering quality, 2) Principal component analysis (PCA) to confirm that t-SNE results aligned with global variance patterns in the dataset, and 3) Machine learning classifiers (e.g., Support Vector Machines, Random Forrest) to evaluate the classification performance of the identified metabolic patterns.

Statistical analysis of cell proliferation, migration, and protein activation, was conducted using a two-tailed independent samples t-test. Each experiment was performed a minimum of three times to ensure reliability. For the analysis of Seahorse data, ANOVA test followed by Bonferroni post hoc analysis was utilized, incorporating at least eight replicates exhibiting consistent trends with minimal variation. Results are presented as the mean standard deviation (SD). The p-values are reported as follows: *p<0.05, **p<0.01, ***p<0.001.
