# Supplementary figures and images for: Oncometabolite signatures from tumor-stroma crosstalk as potential non-invasive biomarkers
Source: Cell Death Discov. 2026 May 22;12:306. doi: 10.1038/s41420-026-03172-1 (PMC13373187; doi:10.1038/s41420-026-03172-1)

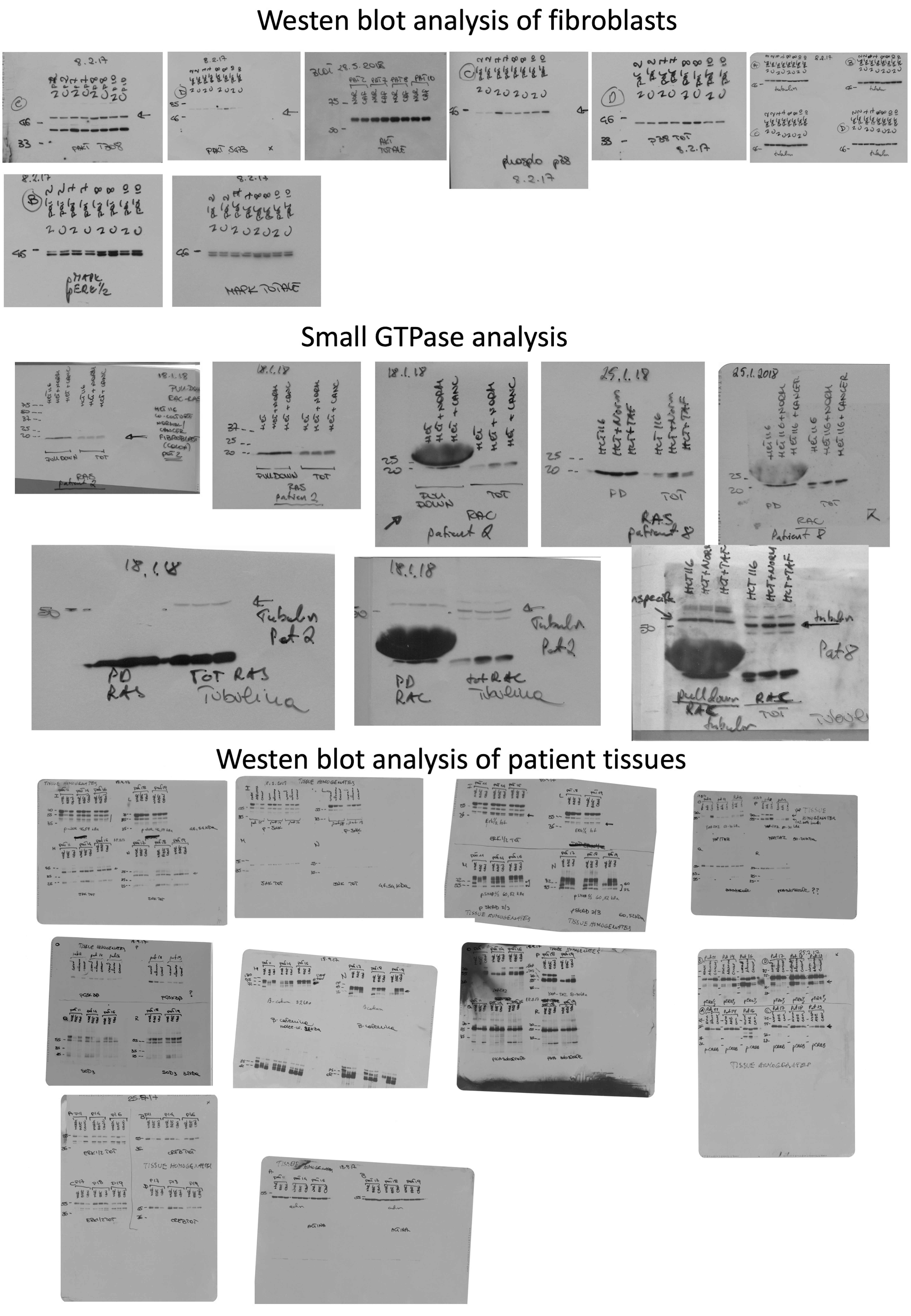

Supplement: Supplementary file 4 — Original Western blots [file 41420_2026_3172_MOESM4_ESM.jpg]
